# The effect of electrode shape on Schottky barrier and electric field distribution of flexible ZnO photodiode

**DOI:** 10.1038/s41598-021-95203-3

**Published:** 2021-08-02

**Authors:** Zahra Aminrayai Jezeh, Babak Efafi, Bijan Ghafary

**Affiliations:** 1 Photonics Lab, Physics Department, Iran University of Science and Technology, Tehran, Iran; 2 Nano Photonics Lab, Applied Science Research Center, Kharazmi University, Alborz, Iran

**Keywords:** Optics and photonics, Physics

## Abstract

In this study, the effect of electrode shape difference on the height of the Schottky barrier and the electric field in flexible photodiodes (PDs) has been investigated. For this purpose, three different electrode designs were prepared on three flexible FR4 layers that were coated with Zinc Oxide (ZnO). The printing circuit board (PCB) method was used to create these copper electrodes. The asymmetry of the PD electrodes and the difference in the height of the Schottky barrier has led to the creation of self-powered PDs. The effect of the amount and shape of the distribution of internal electric fields generated in the PDs and its effect on the parameters of the PDs has been investigated with the help of simulations performed in COMSOL software. The photocurrent of the sample with circular and rectangular electrodes was equal to 470 µA in 15 V bias, which was twice as good as a sample with an interdigitated MSM structure. Also, this sample had the best response time among these three samples, which was equal to 440 ms.

## Introduction

In recent years, the field of flexible and wearable electronic components such as smartwatches, smart glasses and wearable cameras has been growing rapidly. The optical detectors are one of the most important components in wearable electronic devices for measuring light in various applications^1^. Semiconductor materials with wide-bandgap such as ZnO, SiC, GaN, and TiO2 have been used for producing Ultraviolet (UV) PDs and solar cells^2–10^. Among them, ZnO has attracted researchers' attention due to its unique features, including cheapness, high chemical stability, strong radiation hardness, high charge carrier mobility, and most importantly, the 3.37 eV wide-bandgap^3,6,11–14^. Ordinary PDs require an external power supply that increases system power consumption, costs, and system volume. This limits the use of these PDs in cases where access is impossible or dangerous^15^. So far, heterojunction contacts, p-n junctions, and Schottky contacts have been used for producing self-powered PDs^16–23^.

ZnO PDs are mostly based on MSM because they are controllable, stable, and easy to build. Ordinary MSM PDs, as mentioned, needed an external bias source to generate current. Common MSM structures are made up of two symmetrical Schottky contacts that are connected back to back. Studies have been conducted on MSM devices with two different electrodes (one ohmic contact and one Schottky contact), operating at 0 V bias. The production process of these PDs is difficult, and their performance is still not very good. It has recently been shown that the dimensions of electrodes can greatly affect the distribution of the electric field in the Schottky contacts^24^. The electric field can be very effective in separating photogenerated holes and electrons^24^. Thus, a pair of asymmetric electrodes (in terms of material or shape) was used to produce self-powered ZnO MSM PDs^24–27^. The two asymmetry back-to-back Schottky barriers vary in capability to separate and collect the photogenerated electrons and holes, leading to photocurrent organization in the external circuit without external power supply photovoltaic characteristic^15^. Given that many other research groups have worked to improve the parameters of PDs, and for this purpose have used difficult and costly methods that also have the possibility of error, so we were looking for another factor that is effective, easy, and inexpensive that was the effect of electrode shape on Schottky barrier and electric field distribution of PDs.

In this work, three flexible self-powered ZnO MSM samples were prepared using RF sputtering technique on the flexible substrate of FR4 fiberglass. We were fabricated porous ZnO because, according to some studies, porous ZnO can improve the PDs parameters^28–31^. It has been reported that porous ZnO thin films prepared by the unbalanced magnetron sputtering method exhibited a fast UV photoresponse^32^. Using the printed circuit board (PCB) method, electrodes with different geometric shapes for samples 1, 2 and interdigitated electrodes for sample 3 were printed simultaneously. The height of the Schottky barrier at the junction of each electrode was examined using MATLAB software. The effect of the difference between these two Schottky barriers on each PD, which is the basis of self-powered PDs, was so investigated. The Schottky barrier height creates an internal potential and thus leads to the carriers' movement. Therefore, it is an important factor in PD parameters.

Also, the geometric shape of electrodes makes a difference in the shape of the produced electric field and the accumulation of charge carriers that will affect their performance. COMSOL Multiphysics software was used to understand the electrodes' shape on the electric field in these PDs.

Analyses illustrate that sample 1 with two different electrodes in a circular and rectangular shape showed the highest current under illumination in the 0 V bias, which was equal to 0.8 µA, having a high performance compared to the other self-powered PDs UV^24,33^. Also, the amount of the photocurrent is 470 µA at 15 V bias and the response time is 440 ms, which shows a much better performance than similar models of symmetrical electrodes, which is due to the difference in heights of the Schottky barrier, the shape of the produced electric field, and accumulation of charge carriers^26,27,33^.

## Experimental

To prepare the samples, ZnO was coated on the FR4 fiberglass substrate using an RF Sputtering at ambient temperature. The FR4 fiberglass was a flexible substrate with a thickness of 0.15 mm.

ZnO disk with a diameter of 2 in. (99.99%) was used as the target, and the target-substrate distance was 10 cm. During the deposition, the sputtering power was set at 150 W, and the Argon pressure was 20 (mTorr). The deposition time was 1945 s, and the thickness of the ZnO layer deposited on the fiberglass substrate was about 700 nm. The electrodes were printed on the samples using the PCB method. To apply this method, we plotted the shape of the electrodes using Protel software, and the negatives of the design were prepared.

**Figure 1:**
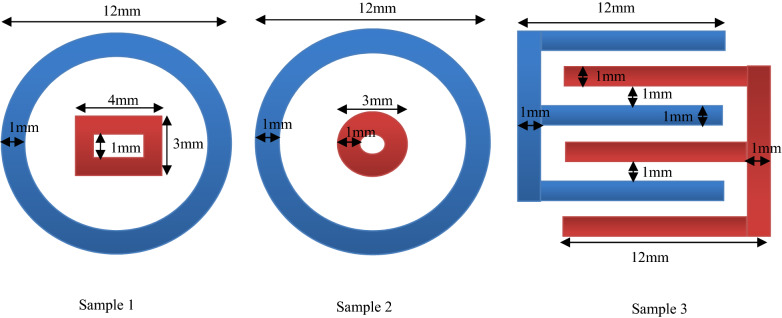
Dimensions of PD electrodes.

Then the negatives were inserted into the PCB machine for printing. These printed electrodes are made of copper with a thickness equal to 35 µm. In this method, hundreds of samples with different electrode shapes can be prepared at the same time. The structure of these PDs was MSM, where the electrodes were provided in different geometric shapes (Fig. 1).

**Figure 2:**
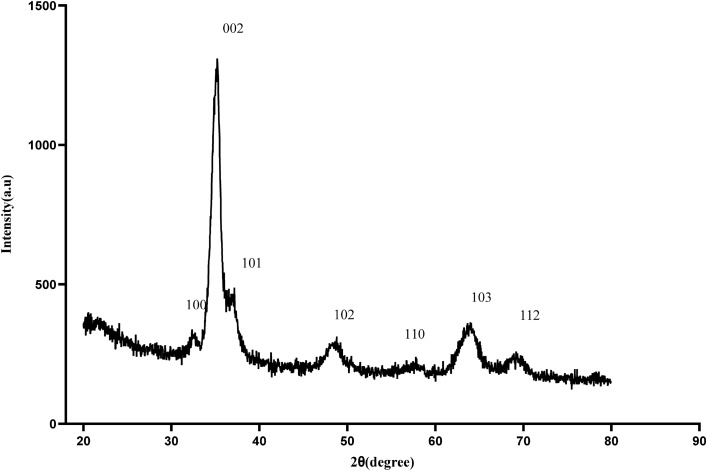
XRD Pattern of flexible UV PDs based on ZnO on the fiberglass (FR4) substrate.

**Figure 3:**
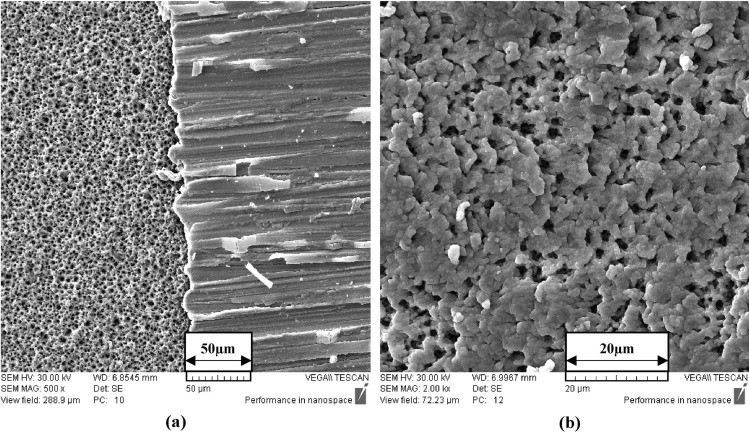
SEM image of flexible UV PDs based on ZnO on the fiberglass (FR4) substrateat **(a)** at × 500 magnification of electrode and ZnO together, **(b)** at × 2 K magnification of ZnO.

The ZnO layer crystalline properties and its morphology were obtained using the X-ray-Diffraction (XRD) (Fig. 2) and the Scanning Electron Microscope (SEM) (Fig. 3).

**Figure 4:**
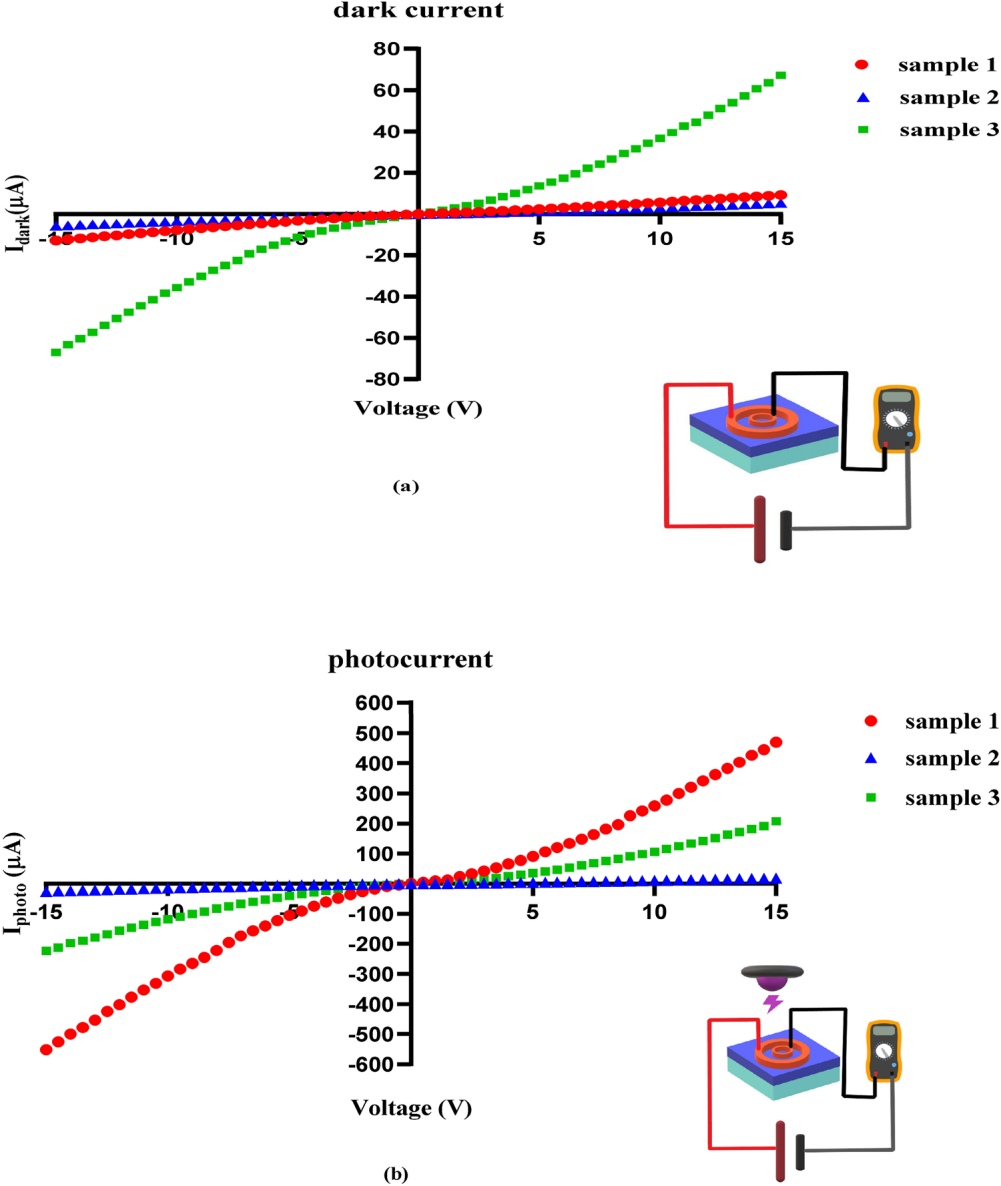
**(a)** Dark current, **(b)** photocurrent of flexible UV PDs based on ZnO on the fiberglass (FR4) substrate.

**Figure 5:**
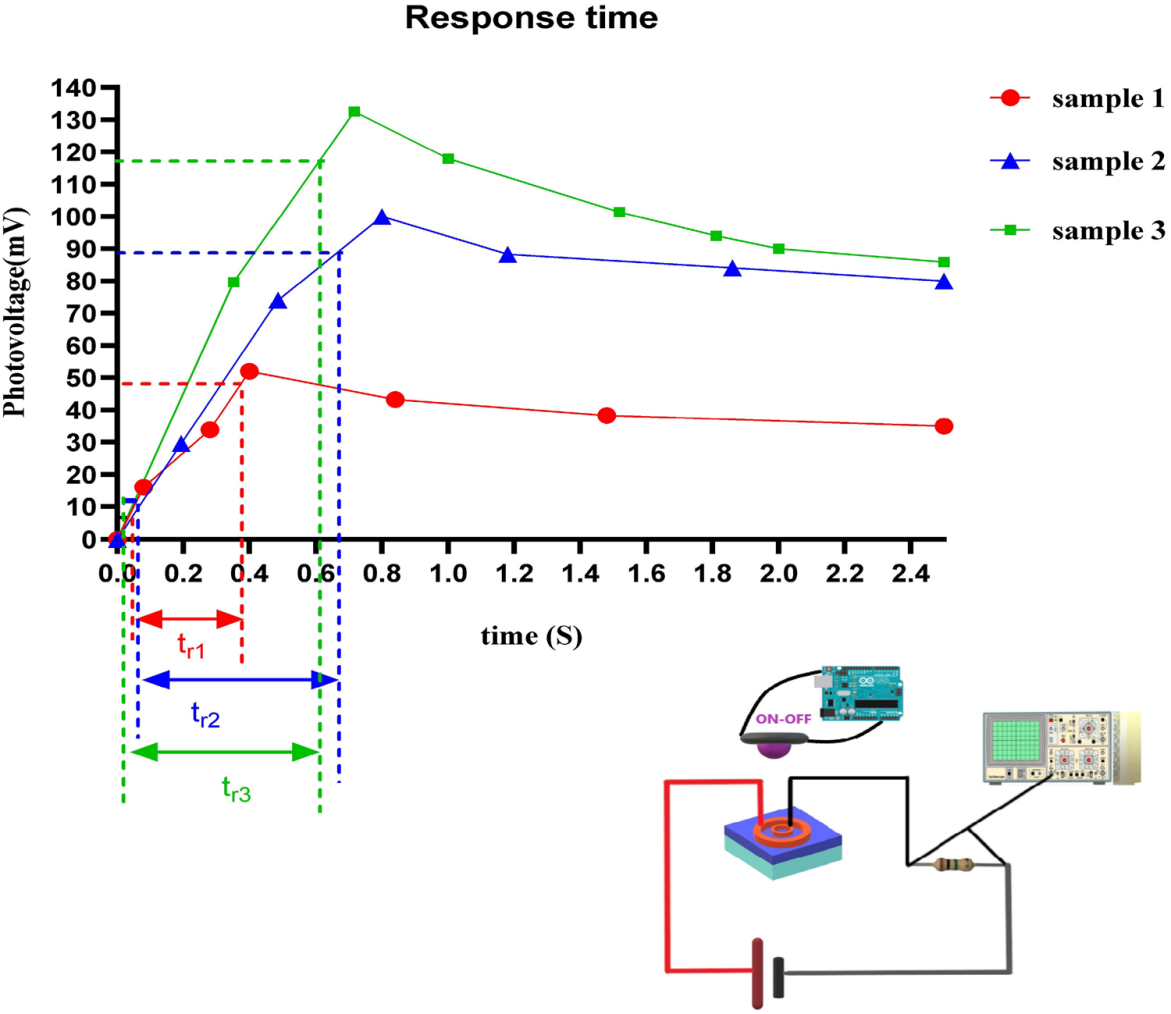
Response time of flexible UV PDs based on ZnO on the fiberglass (FR4) substrate.

Also, the optoelectronic characteristics of the samples, such as dark current (Fig. 4a), photocurrent (Fig. 4b), and response time (Fig. 5) were measured.

## Results and discussion

ZnO thin films' phase pattern is determined using XRD at room temperature with a PANalytical PW3050/60 diffractometer using Cu Ka radiation at 40 kV and 40 mA. The XRD pattern was obtained from phase 2θ, from 20° to 80° with a scan rate of 0.03 °/s. ZnO diffraction peaks are indexed as (100), (002), (101), (102), (110), (103), and (112) for corresponding peak positions of 32.47∘, 35.066∘, 37.02∘,48.46∘, 57.58∘, 63.73∘, and 69.12∘ (Fig. 2). Six hexagonal ZnO peaks can be seen after 30°. The peaks of the spectra correspond to the wurtzite structure of ZnO. The crystal phase identification was performed using ZnO's standard table (JCPDS 65-3411). This film is polycrystalline and has a preferred crystallographic orientation in the (002) plane along the c-axis perpendicular to the substrate surface. The proof for the peak growth in (002) is due to other studies' deposition conditions^34–36^.

SEM images of ZnO's thin layer with the fiberglass substrate show the ZnO layer's porosity (Fig. 3). As can be seen, the porous layer is well-formed. A porous layer is used since other studies have shown that porosity can benefit detector parameters^28–31^.

The work of porous ZnO in the PD can show that the porous vacant in the ZnO layer trap neutral oxygen and increase the response rate due to the neutral oxygen implanted in the grain boundaries in a porous ZnO^32^. The high UV photoelectric response can also be attributed to the high specific surface area. The optoelectronic response performance of ZnO nanomaterials is usually based on their surface state, which causes the upward band to bend close to the surface and trap holes^30,37^. In the dark condition, oxygen molecules absorb ZnO nanomaterials' surface and deplete electrons, creating a thin depletion layer with low electrical conductivity. Electron–hole pairs are created by UV illumination. The holes move to the ZnO surface due to the bending band and discharge of the adsorbed oxygen molecules, leading to the aggregation of electron concentrations and increasing the electrical conductivity. This particular structure increases absorption. This specific surface area also causes response quickly to the applied light illuminated to the ZnO surface.

In other words, the effect of oxygen is that it captures free electrons in dark conditions and trap holes in illumination, increasing the life cycle of photogenerated carriers and improving the photoelectric response performance of ZnO porous films^30^.

In addition to this, in the SEM image, the electrodes and the ZnO layer exist together, which means the electrodes are well arranged on the substrate (Fig. 3a).

Samples 1 and 2 have a special photovoltaic property in 0 V bias due to the electrodes' asymmetry, and both specimens can be called self-powered PDs^15^. To explanation this, the height of the Schottky barrier was calculated^24^. In general, in a Schottky contact, if the E_00_ <  < K_B_T, the thermionic emission overcomes the junction electronic transport process without tunneling, where K_B_ is the constant of Boltzmann, T is absolute temperature, │ E_00_│ is the energy-dependent on the probability of tunneling^38–41^. E_00_ can be calculated using Eq. (1):

1 $$  {\text{E}}_{00} = \left( {{\text{q}}{\hbar}/{2}} \right) \, \left( {{\text{N}}/{\text{m}}^{*} \varepsilon_{{\text{s}}} } \right)^{1/2}  $$

where q is the elementary charge, ћ is the reduced Plank constant, N is the carrier density, m^*^ is the effective mass, and ε_s_ is the relative dielectric permittivity. In this work, m_e_ = 0.27 m_0_, and ε_s_ = 8.3 for ZnO, and the carrier concentration N is about 9.3 × 10^16^ cm^-3^. E_00_ is about 2.2 meV for the ZnO films, much smaller than the thermal energy K_B_T at room temperature (26 meV). Thus, the current passing through the Schottky barrier can be described as follows:

2 $$ {\text{I}} = {\text{I}}_{0} {\text{exp}}\left( {{\text{qv}}/{\text{nK}}_{{\text{B}}} {\text{T}}} \right)\left[ {{ 1} - {\text{exp}}\left( { - {\text{qv}}/{\text{K}}_{{\text{B}}} {\text{T}}} \right)} \right] $$

3 $$ {\text{I}}_{0} = {\text{ AA}}^{*} \, \,{\text{exp}}\left( { - {\text{q}}\phi_{{\text{B}}} /{\text{K}}_{{\text{B}}} {\text{T}}} \right) $$

where K_B_ is the Boltzman constant, T is the absolute temperature, n is the ideality factor, A is the junction area, A^*^ is the Richardson constant (A^*^ = 4пm^*^ q^2^/h^3^), ɸ_B_ is the barrier height, h is the Plank constant, and I_0_ is the reverse saturation current^24^.

The flow passing through the Schottky barrier's height is obtained in a metal–semiconductor contact Eq. (2). Equation (2) can also be rewritten as follows:

4 $$\frac{\mathrm{I}\,\, \exp\left[\mathrm{qV}/\mathrm{kT}\right]}{\mathrm{exp}\left[\mathrm{qV}/\mathrm{kT}\right]-1}={\mathrm{I}}_{0}\,\mathrm{exp}\left[\mathrm{qV}/\mathrm{nkT}\right]$$

**Figure 6:**
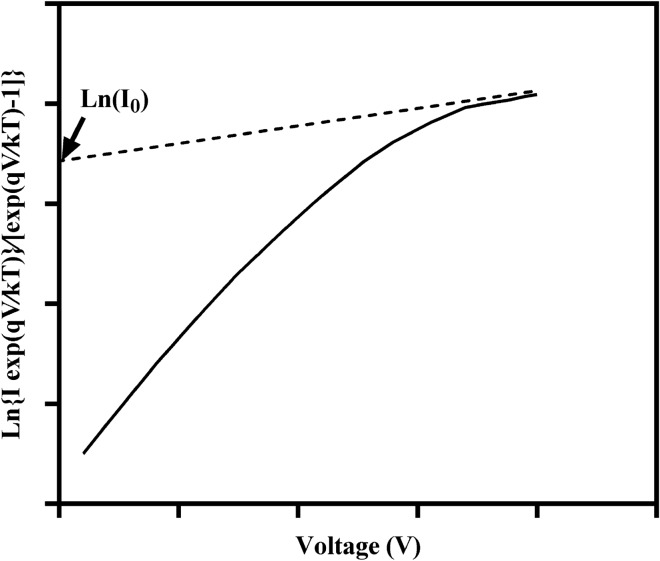
$\mathrm{Ln}\left\{\mathrm{Iexp}\left(\mathrm{qV}/\mathrm{kT}\right)\}/\left[\mathrm{exp}\left(\mathrm{qV}/\mathrm{kT}\right)-1\right]\right\}$vs.V of a MSM PD.

Based on Eq. (4), the plot of Ln $\frac{\mathrm{I}\,\, \exp\left[\mathrm{qV}/\mathrm{kT}\right]}{\mathrm{exp}\left[\mathrm{qV}/\mathrm{kT}\right]-1}$ Vs. V results in a straight line, Ln(I_0_) is derived from the interception with the y-axis (Fig. 6)^42^.

**Figure 7:**
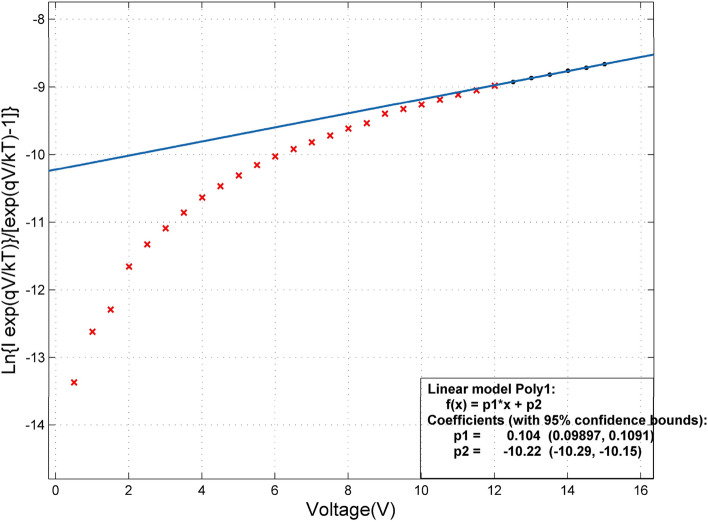
Curve fitting in MATLAB software and obtain Ln (I_0_).

After calculating I_0_ for each connection, the value of I_0_ is placed in Eq. (3), and the height of the Schottky barrier ɸ_B_ for each metal–semiconductor connection is obtained. For each of the PDs, the Schottky barrier's height was obtained corresponding to each one and is given in Table 1. The value of I_0_ was obtained for each electrode-ZnO connection using MATLAB software from diagram of. Figure 7 shows a view of MATLAB software and the calculation of I_0_. P_2_ in Fig. 7 is Ln(I_0_).

**Table 1:** Reverse saturation current, Schottky barrier's height, I _dark_ at 15 V, I _photo_ at 0, 15 V and t_r_ for samples.

Name	I_0_ (A)	ϕBig (eV)	ϕSmall (eV)	I _dark_ at 15 V (µA)	I_photo_15 V (µA)	I_photo_ at 0 V (µA)	t_r_ (ms)
Sample 1	8.232 × 10^–7^	0.651	0.619	9.24	470	0.8	440
Sample 2	4.213 × 10^–7^	0.668	0.624	5.41	18.14	0.3	610
Sample3	4.244 × 10^–6^	0.614	0.614	67.3	208	0	570

**Figure 8:**
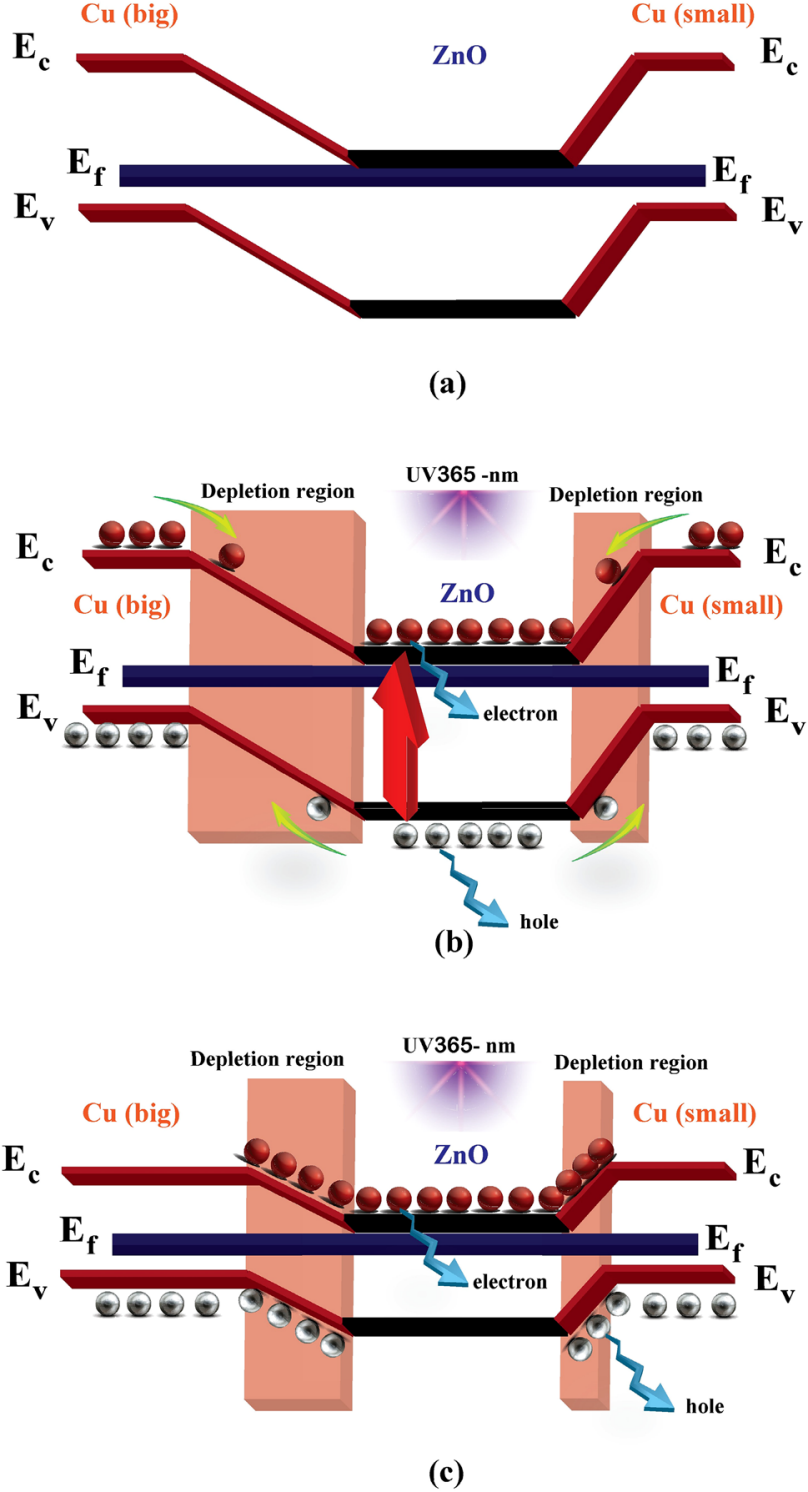
Energy band diagrams of the asymmetric MSM PD at 0 V in the **(a)** dark and **(b,c)** illuminated.

Based on the energy band theory, the energy band diagrams for samples 1,2 under both dark and UV light illuminated conditions are analyzed and illustrated in Fig. 8a and b,c, respectively. In the dark, the Fermi energy levels (E_F_) of ZnO and the electrode are equal. According to the theory calculations^43–45^, the width of the depletion region on the Cu (big)-ZnO interface must be greater than the width of the depletion region on the Cu (small)-ZnO interface. Under ultraviolet light, electron–hole pairs are generated on ZnO's surface, as exhibited in Fig. 8b. The electrons in the conduction band (E_c_) tend to flow away from the metal–semiconductor interfaces, and the holes in the valence band (E_v_) move towards the contact. Collected and trapped holes create a local potential in the interface so that the effective height of the Schottky barrier decreases due to the difference in the width of depletion region between Cu (big)-ZnO and Cu (small)-ZnO, and asymmetric distribution of electric potential in ZnO film can induce the difference in separation and transfer of carrier. Since the number of collected and trapped holes in the two interfaces, which reduces the height of the Schottky barrier between two electrodes of Cu (big)-ZnO and Cu (small)-ZnO, which because the number of holes in The Cu (big) -ZnO interface is larger, so the Schottky barrier height reduction is greater (Fig. 8c)^46^. As a result, typical photovoltaic specifications can be seen in asymmetric MSM PDs at a bias voltage of 0 V (samples 1,2). It is worth noting that the change in the height of the barrier is highly dependent on ZnO's electrical properties and the Cu electrodes' structure, which can determine the local potential^16,24,33,38,47–50^.

When a bias voltage is applied between two electrodes, an electric field is created and causes the charge carriers to move, which leads to the production of current. The difference in the electrodes' shape leads to the difference in the electric field's shape and amount. As a result, the dark current (Fig. 4a), photocurrent (Fig. 4b) and response time (Fig. 5) also vary. To show the influence of an electric field in the organization of electric charges, the electric displacement field is defined^51^:

5 $$ {\text{D}} = \varepsilon_{0} \varepsilon_{{\text{r}}} {\text{E}} $$

where ε_0_ is the vacuum permittivity, ε_r_ is the relative (dielectric) permittivity, and E is the electric field. The question arises that we do not have the values ε_0_ and ε_r_. What should we do? The answer is that since ε_0_ and ε_r_ are related to the material, and all three of our samples are the same material, therefore ε_0_ and ε_r_ are the same for all three of our samples, And because our work is comparative, the amount does not matter to us. Based on Eq. (5), the difference in E leads to the difference in D.

We used COMSOL Multiphysics software to obtain the electric displacement field, electric field, and total electric energy. As mentioned, the amount of current caused by charge carriers' movement is directly related to the electric field (E) and the electric displacement field (D).

**Figure 9:**
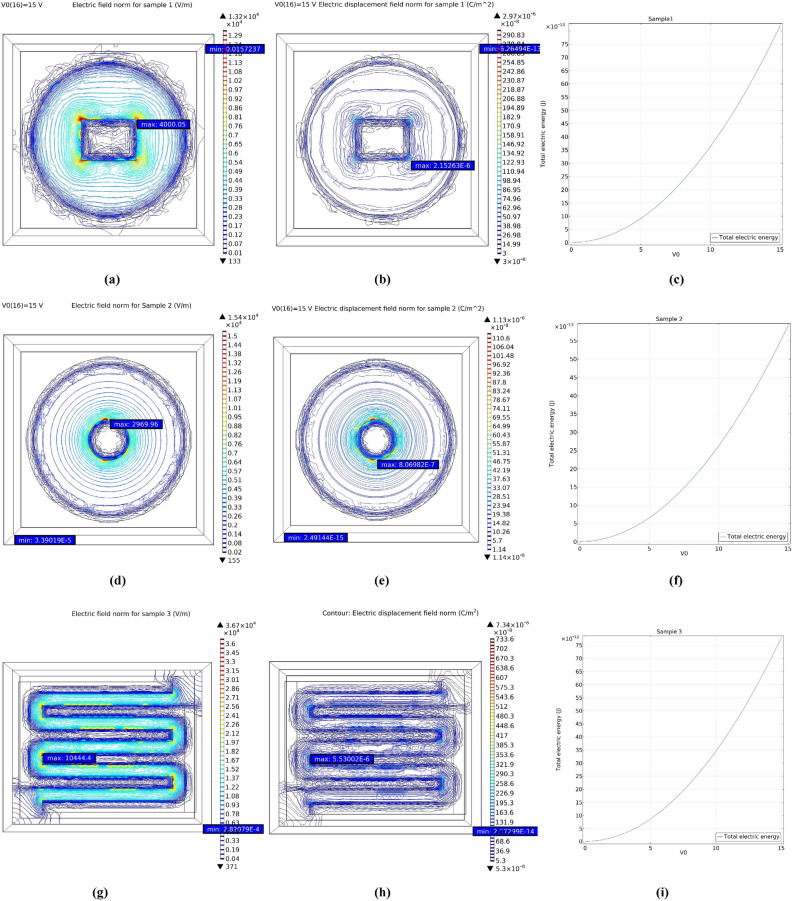
Simulation in COMSOL multiphysics software **(a)** electric field norm for sample 1 at 15 V bias, **(b)** electric displacement field norm for sample 1 at 15 V bias, **(c)** total electric energy for sample 1 at 0 to 15 V bias, **(d)** electric field norm for sample 2 at 15 V bias, **(e)** electric displacement field norm for sample 2 at 15 V bias, **(f)** total electric energy for sample 2 at 0 to 15 V bias, **(g)** electric field norm for sample 3 at 15 V bias, **(h)** electric displacement field norm for sample 3 at 15 V bias, **(i)** total electric energy for sample 3 at 0 to 15 V bias.

The electric field and electric displacement field simulation results at 15 V bias and total electric energy are given for all three samples (Fig. 9). Electric current results from electric charge movement around a circuit, but to move an electric charge from one electrode to another, there needs to be a force to create the work to move the electric charge. The total electric energy is defined:

6 $$ {\text{J}} = {\text{V}}.{\text{C}} $$

J is the total electric energy, V is the voltage, and C is the electric charge.

The total electric energy at the same voltage for these samples was as follows (Fig. 9c,f,i):

$$ {\text{J}}_{{2}} < {\text{ J}}_{{1}} < {\text{ J}}_{{3}} $$

J (J) in 15 V are:

$$ {58} \times {1}0^{{ - {13}}} < {83} \times {1}0^{{ - {13}}} < { 77} \times {1}0^{{ - {12}}} $$

Based on Eq. (6), at the same voltage, we have:

$$ {\text{C2 }} < {\text{ C1 }} < {\text{ C3}} $$

As seen in Fig. 9, The order of the maximum of the E (V/m) and the D (C/m^2^) at bias 15 V are as follows:

$$ {\text{E}}_{2} \left( 2969.96 \right) \, < {\text{ E}}_{1} \left( 4000.05 \right) \, < {\text{ E}}_{3} \left( 10444.4 \right) $$

$$ {\text{D}}_{2} \left(8.06 \times {1}0^{- 7 } \right) \, < {\text{ D}}_{1} \left( 2.15 \times 10^{ - 6} \right) \, < {\text{ D}}_{3} \left(5.53 \times 10^{ - 6 } \right) $$

In the dark, the issue of the Schottky barrier and local potential difference is not raised. In Table 1, dark current (µA) at bias 15 V:

$$ {\text{I}}_{{{\text{D2}}}} \left( {{5}.{41}} \right) \, < {\text{ I}}_{{{\text{D1}}}} \left( {{9}.{24}} \right) \, < {\text{ I}}_{{{\text{D3}}}} \left( {{67}.{3}} \right) $$

In general, current means the movement of charge carriers regardless of what is due. According to the statements, the greater the electric displacement field that causes the carriers to move, the greater the current.

As a result, it can be seen that the simulation confirms our experimental data. Under illuminated, in addition to the electric field, the local electric potential, which is caused by the electrodes' asymmetry, is also involved. As a result, these two factors must be considered together. Our experimental results, the highest electric current under illuminated in sample 1, exceeded it in sample 3. As we learn from the basic concepts of electromagnetism, the electric field is more intense at the sharp points. As a result, the electric field's intensity in those areas is more intense can see in Fig. 9a,b; at the corners of the rectangular electrode, the density of electric field lines is higher. As a result of both the shape and the amount of electric field, as well as the asymmetry of the electrodes and the creation of a local potential difference, illuminated current (µA) at bias 15 V for PDs are:

$$ {\text{I}}_{{{\text{illu2}}}} \left( {{18}.{14}} \right) \, < {\text{ I}}_{{{\text{illu3}}}} \left( {{2}0{8}} \right) \, < {\text{ I}}_{{{\text{illu1}}}} \left( {{47}0} \right) $$

The local potential difference due to the electrodes' asymmetry creates an electric field that helps compensate for the smaller electric field amount in sample 1 compared to sample 3. As a result of these two factors, sample 1 surpass sample 3. In sample 2, because its field is much smaller than samples 1 and 3 (therefore, it has the lowest amount of dark current) and its local potential difference due to the electrodes' asymmetry could not compensate, so it is observed. Which also has the lowest amount of current under illuminated.

Also, the responsivity of a PD provides information related to the generation of a photocurrent per unit incident UV power on a PD. Moreover, the specific detectivity defines the information associated with the ability to detect a weak UV illumination by a PD. In general, the specific detectivity of a PD is related to its noise. The responsivity and specific detectivity were measured as follows^52^:

$$ {\text{R}} = \frac{{{\text{photocurrent}}}}{{\text{incident optical power}}}\quad \left( {{\text{AW}}^{{ - {1}}} } \right) $$

$$ {\text{Specific detectivity}} = {\text{D}}^{*} = {\text{R}}_{{\text{s}}} \times \left( {\frac{{{\text{A}}_{0} }}{{2{\text{eI}}_{{{\text{dark}}}} }}} \right)^{\frac{1}{2}} \quad \left( {{\text{Jones}}} \right) $$

where e is the absolute value of elementary charge and A_o_ is the UV signal exposed area.

The responsivity for samples 1,2 and 3 in the wavelength 365 nm is equal to, respectively 1.2, 0.05 and 0.53 AW^-1^ at a bias voltage of 15 V. Also, specific detectivity for them in the same conditions is equal to, respectively 5.77 × 10^11^, 3.2 × 10^10^ and 9.5 × 10^10^ Jones. These data also show the superiority of sample 1 among other samples.

For response time, photogenerated carriers move faster with a drift speed due to the electric field, which improves the photon flow and causes higher response speeds. The point is that for the response time because there is illuminated, due to the Schottky barrier and the local potential difference is also involved, the response time (ms) at bias 5 V for these PDs is:

$$ {\text{t}}_{{{\text{r1}}}} \left( {{44}0} \right) \, < {\text{ t}}_{{{\text{r3}}}} \left( {{57}0} \right) \, < {\text{ t}}_{{{\text{r2}}}} \left( {{61}0} \right) $$

In short, if only the difference between the Schottky barrier is considered, sample 2, sample 1, and finally sample 3, respectively, had the largest difference (using MATLAB software). If only the shape and amount of electric field are considered, the maximum value should be for sample 3, then sample 1, and finally sample 2 (with COMSOL multiphysics software). While both of these factors must be considered together, and when both are considered together, it can be seen that in sample 1, a difference in the Schottky barrier leads to local potential and thus to a local electric field. It can be prepared with the help of the main electric field and surpass the photocurrent of sample 1 from sample 3. In sample 2, because the electric field amount was much lower, this local electric field could not compensate for this shortage.

## Conclusion

In this study, the effect of electrode shape on the parameters of flexible UV PDs based on porous ZnO on the fiberglass (Fr4) substrate was investigated. It was observed that the difference in the height of the Schottky barrier could be the basis for the creation of self-powered PDs. Also, the shape of the electrodes affects the amount and shape of the electric field created, which is the transfer factor of the charge carriers, which leads to a change in the output current of the PDs. Among these 3 PDs, which were fabricated simultaneously using RF sputtering and PCB techniques, sample 1 was the best in the current at 0 V (0.8 µA), the photocurrent (at 15 V = 470µA) and the response time (440 ms). From experimental data and information obtained from computing and simulation software (MATLAB and COMSOL Multiphysics), it can be concluded that the output currents of the PDs in illuminated are related to the difference of potentials and electric fields created. These samples are different in two ways: first, the difference in local potential due to the difference in the height of the Schottky barrier, and second, the difference in shape and amount of electric field due to the difference in the shape of the electrodes. Between these 3 PDs, sample 1 with two circular and rectangular electrodes showed the best performance under illuminated conditions (both at 0 V and in bias mode).

## References

[1] Pei Y, Pei R, Liang X, Wang Y, Liu L, Chen H, Liang J (2016). CdS-nanowires flexible photo-detector with Ag-nanowires electrode based on non-transfer process. Sci. Rep..

[2] Gao J, Liu W-J, Ding S-J, Lu H-L, Zhang DW (2018). High performance ultraviolet photodetectors with atomic-layer-deposited ZnO films via low-temperature post-annealing in air. AIP Adv..

[3] Efafi B, Mazandarani H, Ara MHM, Ghafary B (2020). Improvement in photoluminescence behavior of well-aligned ZnO nanorods by optimization of thermodynamic parameters. Phys. B Condens. Matter.

[4] Efafi B, Mousavi SS, Majlesara MH, Ghafary B, Sajad B (2018). Fabrication of high-performance UVC photodiodes by Al+ 3 ion adjustment in AZO/Si Heterostructures. Opt. Mater..

[5] Mousavi SS, Kazempour A, Efafi B, Ara MHM, Sajad B (2019). Effects of graphene quantum dots interlayer on performance of ZnO-based photodetectors. Appl. Surf. Sci..

[6] Efafi B, Mousavi SS, Ara MHM, Ghafari B, Mazandarani HR (2017). A method for optimizing the electrical conductivity of Al: ZnO TCO films. Mater. Lett..

[7] Efafi B, Ghamsari MS, Aberoumand M, Ara MM, Ghamsari AS, Rad HH (2014). Aluminum doped ZnO sol–gel derived nanocrystals: Raman spectroscopy and solid solubility characterization. Phys. Status Solidi (A).

[8] Upadhyay GK, Kumar V, Purohit L (2020). Optimized CdO: TiO2 nanocomposites for heterojunction solar cell applications. J. Alloys Compds..

[9] Ozcan C, Turkay D, Yerci S (2019). Optical and electrical design guidelines for ZnO/CdS nanorod-based CdTe solar cells. Opt. Express.

[10] Taleghani SS, Meymian MRZ, Ameri M (2016). Interfacial modification to optimize stainless steel photoanode design for flexible dye sensitized solar cells: an experimental and numerical modeling approach. J. Phys. D Appl. Phys..

[11] Liu K, Sakurai M, Aono M (2010). ZnO-based ultraviolet photodetectors. Sensors.

[12] Mousavi SS, Sajad B, Efafi B, Alaibakhsh H, Jahromi KE, Majlesara MH, Ghafary B (2018). Practical optimization of highly sensitive azo photoconductor with circular electrode scheme. J. Lightwave Technol..

[13] Pathak TK, Kumar V, Swart H, Purohit L (2015). P-type conductivity in doped and codoped ZnO thin films synthesized by RF magnetron sputtering. J. Mod. Opt..

[14] Ameri M, Raoufi M, Zamani-Meymian M-R, Samavat F, Fathollahi M-R, Mohajerani E (2018). Self-assembled ZnO nanosheet-based spherical structure as photoanode in dye-sensitized solar cells. J. Electron. Mater..

[15] An Q, Meng X, Xiong K, Qiu Y (2017). Self-powered ZnS nanotubes/Ag nanowires MSM UV photodetector with high on/off ratio and fast response speed. Sci. Rep..

[16] Liu K, Shen D, Shan C, Zhang J, Yao B, Zhao D, Lu Y, Fan X (2007). Zn 0.76 Mg 0.24 O homojunction photodiode for ultraviolet detection. Appl. Phys. Lett..

[17] Zhu H, Shan C, Yao B, Li B, Zhang J, Zhao D, Shen D, Fan X (2008). High spectrum selectivity ultraviolet photodetector fabricated from an n-ZnO/p-GaN heterojunction. J. Phys. Chem. C.

[18] Bie YQ, Liao ZM, Zhang HZ, Li GR, Ye Y, Zhou YB, Xu J, Qin ZX, Dai L, Yu DP (2011). Self-powered, ultrafast, visible-blind UV detection and optical logical operation based on ZnO/GaN nanoscale p-n junctions. Adv. Mater..

[19] Shaikh PA, Thakare VP, Late DJ, Ogale S (2014). A back-to-back MOS–Schottky (Pt–SiO 2–Si–C–Pt) nano-heterojunction device as an efficient self-powered photodetector: One step fabrication by pulsed laser deposition. Nanoscale.

[20] Mandal L, Chaudhari NS, Ogale S (2013). Self-powered UV-vis photodetector based on ZnIn2S4/hydrogel interface. ACS Appl. Mater. Interfaces..

[21] Wang Z, Ran S, Liu B, Chen D, Shen G (2012). Multilayer TiO 2 nanorod cloth/nanorod array electrode for dye-sensitized solar cells and self-powered UV detectors. Nanoscale.

[22] Li X, Gao C, Duan H, Lu B, Pan X, Xie E (2012). Nanocrystalline TiO2 film based photoelectrochemical cell as self-powered UV-photodetector. Nano Energy.

[23] Xie Y, Wei L, Wei G, Li Q, Wang D, Chen Y, Yan S, Liu G, Mei L, Jiao J (2013). A self-powered UV photodetector based on TiO 2 nanorod arrays. Nanoscale Res. Lett..

[24] Chen H-Y, Liu K-W, Chen X, Zhang Z-Z, Fan M-M, Jiang M-M, Xie X-H, Zhao H-F, Shen D-Z (2014). Realization of a self-powered ZnO MSM UV photodetector with high responsivity using an asymmetric pair of Au electrodes. J. Mater. Chem. C.

[25] Li D, Sun X, Song H, Li Z, Jiang H, Chen Y, Miao G, Shen B (2011). Effect of asymmetric Schottky barrier on GaN-based metal-semiconductor-metal ultraviolet detector. Appl. Phys. Lett..

[26] Park J-H, Yu H-Y (2011). Dark current suppression in an erbium–germanium–erbium photodetector with an asymmetric electrode area. Opt. Lett..

[27] Peng M, Liu Y, Yu A, Zhang Y, Liu C, Liu J, Wu W, Zhang K, Shi X, Kou J (2016). Flexible self-powered GaN ultraviolet photoswitch with piezo-phototronic effect enhanced on/off ratio. ACS Nano.

[28] Liu S, Sakai M, Liu B, Terashima C, Nakata K, Fujishima A (2013). Facile synthesis of transparent superhydrophobic titania coating by using soot as a nanoimprint template. RSC Adv..

[29] Liu H, Ye T, Mao C (2007). Fluorescent carbon nanoparticles derived from candle soot. Angew. Chem..

[30] Cao Y, Deng S, Hu Q, Zhong Q, Luo Q-P, Yuan L, Zhou J (2015). Three-dimensional ZnO porous films for self-cleaning ultraviolet photodetectors. RSC Adv..

[31] Ismail RA (2010). Fabrication and characterization of photodetector based on porous silicon. J. Surf. Sci. Nanotechnol..

[32] Sharma P, Mansingh A, Sreenivas K (2002). Ultraviolet photoresponse of porous ZnO thin films prepared by unbalanced magnetron sputtering. Appl. Phys. Lett..

[33] Dong L, Yu J, Jia R, Hu J, Zhang Y, Sun J (2019). Self-powered MSM deep-ultraviolet β-Ga 2 O 3 photodetector realized by an asymmetrical pair of Schottky contacts. Opt. Mater. Exp..

[34] Zoolfakar AS, Rani RA, Morfa AJ, Balendhran S, O'Mullane AP, Zhuiykov S, Kalantar-zadeh K (2012). Enhancing the current density of electrodeposited ZnO–Cu 2 O solar cells by engineering their heterointerfaces. J. Mater. Chem..

[35] Rajan, L., Periasamy, C., & Sahula, V. Structural and optical characteristics of RF sputtered ZnO thinfilm on Si substrate for device applications. In *2015 Annual IEEE India Conference (INDICON)* 1–4. 10.1109/INDICON.2015.7443410, (IEEE, 2015).

[36] Singh J, Ranwa S, Akhtar J, Kumar M (2015). Growth of residual stress-free ZnO films on SiO2/Si substrate at room temperature for MEMS devices. AIP Adv..

[37] Djurišić A, Ng AMC, Chen X (2010). ZnO nanostructures for optoelectronics: Material properties and device applications. Prog. Quantum Electron..

[38] Sze SM (2008). Semiconductor Devices: Physics and Technology.

[39] Nie B, Hu JG, Luo LB, Xie C, Zeng LH, Lv P, Li FZ, Jie JS, Feng M, Wu CY (2013). Monolayer graphene film on ZnO nanorod array for high-performance Schottky junction ultraviolet photodetectors. Small.

[40] Liu J, Shan C, Li B, Zhang Z, Yang C, Shen D, Fan X (2010). High responsivity ultraviolet photodetector realized via a carrier-trapping process. Appl. Phys. Lett..

[41] Xie X, Zhang Z, Li B, Wang S, Jiang M, Shan C, Zhao D, Chen H, Shen D (2014). Enhanced solar-blind responsivity of photodetectors based on cubic MgZnO films via gallium doping. Opt. Express.

[42] Jandow N, Hassan HA, Yam F, Ibrahim K (2012). ZnO metal-semiconductor-metal UV photodetectors on PPC plastic with various metal contacts. Photodetectors.

[43] Freeouf J, Jackson T, Laux S, Woodall J (1982). Effective barrier heights of mixed phase contacts: Size effects. Appl. Phys. Lett..

[44] Smit G, Rogge S, Klapwijk T (2002). Scaling of nano-Schottky-diodes. Appl. Phys. Lett..

[45] Donolato C (2004). Approximate analytical solution to the space charge problem in nanosized Schottky diodes. J. Appl. Phys..

[46] Chen H, Sun X, Yao D, Xie X, Ling F, Su S (2019). Back-to-back asymmetric Schottky-type self-powered UV photodetector based on ternary alloy MgZnO. J. Phys. D Appl. Phys..

[47] Wang L, Ju Z, Zhang J, Zheng J, Shen D, Yao B, Zhao D, Zhang Z, Li B, Shan C (2009). Single-crystalline cubic MgZnO films and their application in deep-ultraviolet optoelectronic devices. Appl. Phys. Lett..

[48] Look DC (2001). Recent advances in ZnO materials and devices. Mater. Sci. Eng. B.

[49] Rogers DL (1991). Integrated optical receivers using MSM detectors. J. Lightwave Technol..

[50] Zheng Q, Huang F, Ding K, Huang J, Chen D, Zhan Z, Lin Z (2011). MgZnO-based metal-semiconductor-metal solar-blind photodetectors on ZnO substrates. Appl. Phys. Lett..

[51] Masouleh, F.F. & Das, N. Application of metal-semiconductor-metal photodetector in high-speed optical communication systems. In *InTech2014*, 10.5772/58997, (2014).

[52] Boruah BD (2019). Zinc oxide ultraviolet photodetectors: Rapid progress from conventional to self-powered photodetectors. Nanoscale Adv..

